# Ethnopharmacological Survey on Medicinal Plants Used for Cosmetic Treatments in Traditional and Ayurveda Systems of Medicine in Sri Lanka

**DOI:** 10.1155/2021/5599654

**Published:** 2021-06-26

**Authors:** Dehel Gamage Nadeeshani Dilhara Gamage, Rathnayaka Mudiyanselage Dharmadasa, Don Chandana Abeysinghe, Rathnayaka Gamlathge Saman Wijesekara, Gamika A. Prathapasinghe, Takao Someya

**Affiliations:** ^1^Faculty of Agriculture and Plantation Management, Wayamba University of Sri Lanka, Makandura, Gonawila. 60170, Sri Lanka; ^2^Industrial Technology Institute, 363, Bauddhaloka Mawatha, Colombo 7, Sri Lanka; ^3^Faculty of Livestock, Fisheries and Nutrition, Wayamba University of Sri Lanka, Makandura, Gonawila. 60170, Sri Lanka; ^4^ALBION Co.,Ltd, Ginza 1-7-10, Chuo-ku, Tokyo 104-0061, Japan

## Abstract

Medicinal plants have been used for therapeutic and beauty applications in Sri Lanka with documented history of over 2,500 years. This inherited knowledge, which has been handed down from generation to generation, provides a largely unexplored source for the potential development of active ingredients for cosmetic formulations. Therefore, the present comprehensive survey was conducted to identify cosmetic potential medicinal plants species in Sri Lanka. Personal interviews were conducted via a semistructured questionnaire with randomly selected 30 traditional practitioners and 90 Ayurveda physicians in Sri Lanka. Data were collected on plants and specific plant parts used for the treatment of skin care, hair care, and oral care topically. The acquired data were verified using the Ayurveda authentic books and quantitatively analyzed using relative frequency of citation (RFC), use value (UV), relative importance (RI), and factor informant consensus (FIC). Results revealed about the usage of 133 different plant species belonging to 64 families in cosmetic treatments under the categories of skin care, hair care, and oral care. Majority of medicinal plants were used in skin care treatments (39%) followed by hair care (20%) and oral care (17%). *Aloe vera* (L.) Burm.f. reported the highest RFC value (0.83) and UV (3.66). The highest RI value was reported from *Asparagus racemosus* Willd. and *Hibiscus rosa-sinensis* L. (1.67). The dominant plant family was reported as family Fabaceae. The most utilized plant part was stated as leaves (34%) followed by bark (14%). The survey further revealed about treatments for 17 skin-related, 9 hair-related, and 2 oral-related beauty issues. All RFC values were comparatively high for identified different beauty issues. Many herbal preparations were prepared using water as the medium whilst most common mode of application was reported as paste (37%). In conclusion, acquired information could ultimately be utilized for the development of the herbal cosmetic industry through the isolation and characterization of bioactive compounds from the documented plants while preserving the traditional knowledge.

## 1. Introduction

Sri Lanka, formerly known as Ceylon, is an island with an area of approximately 65,610 km^2^. Despite its relatively small size, Sri Lanka possesses a high level of biodiversity due to its varied climate and topographical conditions [1]. As one of the most biologically diverse countries in Asia, Sri Lanka currently has 29.7% of forest cover [2] and 4,143 plant species distributed within 214 families. Of these, 1,025 plant species are endemic to the country [3]. In view of that, Sri Lanka is recognized as a biodiversity hotspot of global and national importance.

Plants have been used for treating various illnesses over thousand years through four systems of traditional medicine in Sri Lanka called *Ayurveda*, *Siddha*, *Unani*, and *Deshiya Chikitsa* [4]. Traditional systems of medicine play a pivotal role in lives of Sri Lankan rural population by fulfilling 60–70% of primary health care needs [5]. According to the Sugathadasa et al. [6], 1,430 species representing 181 families and 838 genera are considered as medicinal plants. Out of the total number of medicinal plant species, 174 (12%) are endemic to the country. As described by Pushpakumara et al. [1], 250 species of medicinal plants are commonly used in traditional medicine of which 50 species are heavily used. Thus, it clearly implies the importance of medicinal plants in different systems of medicine in Sri Lanka.

Medicinal plants have been used for centuries in medicinal, therapeutic, and beauty applications in Sri Lanka, which has documented history of over 2,500 years. However, many formulae for medicinal preparations of Sri Lankan traditional system of medicine are handed down from generation to generation or are found only in the scripts of old “ola leaf” books treasured by traditional and Ayurvedic practitioners [7]. Furthermore, the study on “Medicinal Plant Research in Sri Lanka: A Scientometric Study Based on Scopus Database,” highlights about the research studies of 190 plants including 22 endemic plants. It reveals that most of the conducted studies are activity-based studies such as toxicity, antibacterial, antifungal, hypoglycemic, antioxidant, anti-inflammatory, and diuretic. This is followed by general studies such as physicochemical, chemical, postharvest, horticultural, and propagation studies of plants [8]. These provide evidence for a largely unexplored knowledge gap of medicinal plants in Sri Lanka.

Apart from the studies on “Cosmetic Perspective of Ethnobotany in Northern Part of Sri Lanka” [9], there has been hardly any ethnobotany report on cosmetic potential of Sri Lankan medicinal plants. A total of 62 plant species belonging 36 families have been identified based on the traditional knowledge and practices of local community through this study. Identified plants are used for beautifying purposes such as skin care, hair care, nail care, lip care, and eye care. Recently conducted few handfuls of research studies highlight about several medicinal plants with cosmetic potential. Napagoda et al. [10] highlights the probable usage of *Atalantia ceylanica* (Arn.) Oliver, *Hibiscus furcatus* Mullend., *Leucas zeylanica* (L.) W.T.Aiton, *Mollugo cerviana* (L.) Ser., *Olax zeylanica* L., and *Ophiorrhiza mungos* L. for the development of photoprotective cosmetic products via analyzing antioxidant activity and the sun protection factor (SPF). Moreover, research study conducted by Liyanaarachchi et al. [11] emphasizes about possible usage of *Artocarpus nobilis* Thw., *Artocarpus altilis* (Parkinson) Fosberg, *Elaeocarpus serratus* Heyne, *Curcuma aromatica*, and *Artocarpus heterophyllus* Lam. in the treatment of various skin disorders such as hyperpigmentation, to obtain lighter skin complexion, wrinkling, premature aging, and biological aging by analyzing tyrosinase, elastase, and hyaluronidase enzyme inhibitory and antioxidant activities.

Nevertheless, there are only handfuls of scientific evidence available on bioactivity studies of medicinal plants in Sri Lanka that could lead to the development of herbal cosmetics [10]. Thus, it is a necessary requirement of the country to investigate the medicinal plants which exhibit cosmetic potential to support herbal cosmetic productions, innovations, and bioactivity studies while preserving the existing knowledge. In order to fulfil this knowledge gap, the present survey has focused to identify plants and plant parts used in topical cosmetic treatments of Sri Lanka under the categories of skin care, hair care, and oral care by interviewing traditional practitioners and Ayurveda physicians. Furthermore, major beauty issues treated by practitioners and mode of treatment applications have also been studied. Thereby, we believe the present study will support the development of the herbal cosmetic industry of Sri Lanka while rationalizing the ethnopharmacological usage.

## 2. Materials and Methods

### 2.1. Study Area and Selection of Respondents

**Figure 1:**
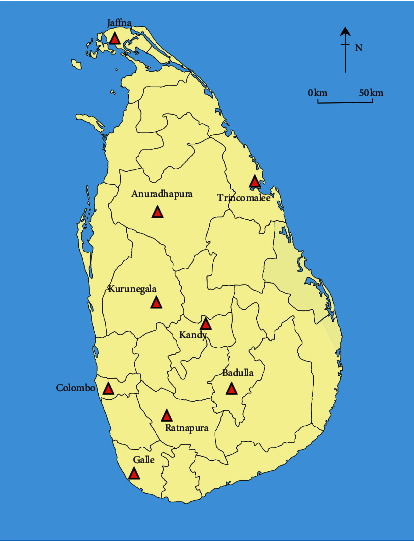
Locations of the provincial Ayurveda Departments in Sri Lanka.

This survey was conducted from January 2018 to August 2019. Ayurveda physicians and traditional practitioners engaged in cosmetic treatments were only considered. Names of the expert practitioners were listed by visiting the Ayurvedic Medical Council and from 09 provincial Ayurveda Departments (Northern Province, North Western Province, Western Province, Central Province, Uva Province, Sabaragamuwa Province, Eastern Province, Southern Province, and North Central Province), Sri Lanka (Figure 1). In total, 210 expert practitioners (163 Ayurveda physicians and 47 traditional practitioners) were identified. Prior to data collection, each respondent was informed of the objectives of the study in order to obtain their consent and cooperation for the survey. However, permission was received only from 90 Ayurveda physicians and 30 traditional practitioners (Table 1). The total respondent percentage was 57%.

### 2.2. Preparation of Questionnaire

Firstly, the questionnaire (Supplementary Material 1) was pretested by interviewing 20 practitioners prior to the formal survey to understand the effectiveness of the questionnaire. Information was collected via a semistructured questionnaire under two main sections. The first section was designed to gather general information about the practitioners including practitioner's name, gender, age, experience of the profession, level of education, address, province/district, and registration number assigned by the Ayurveda Department of Sri Lanka.

The second section of the questionnaire was mainly dedicated to gather the information on medicinal plants and plant parts used in cosmetic treatments. Major beauty issues treated by practitioners and application mode of treatments were also studied. However, information on cosmetic remedies was not collected due to the unwillingness of practitioners to disclose their family recipes. In addition, suggestions for improving the research based on medicinal plants in cosmetics were recorded. Data collection was done by personal interviews.

### 2.3. Taxonomical Studies and Plant Specimens

Collected plants/plant materials from the practitioners were dried, preserved, and mounted on herbarium sheets. Herbarium voucher numbers were coded from NGHC 01 to NGHC 133 as the same order of the plant list indicated in Table 2. Plant identification was carried out by comparing the deposited herbarium of the royal botanical garden, Peradeniya, Sri Lanka. The scientific names of plants were validated based on the collections listed in the homepage: http://www.theplantlist.org. The acquired data including vernacular names, English names, and remedies were verified using the Ayurveda authentic books, “Compendium of Medicinal Plants, A Sri Lankan Study,” volume I to IV issued by Ayurveda Department of Sri Lanka [12–15] and “A Collection of Medicinal Plants in Sri Lanka,” issued by Nature's Beauty Creations Limited, Sri Lanka [16].

### 2.4. Quantitative Analysis of Information

The traditional knowledge of medicinal plant usage in cosmetic treatments was quantitatively analyzed using the relative frequency of citation, use value, factor informant consensus, and relative importance as standard methods described by Hoffman and Gallaher, [17] and Vitalini et al. [18].

#### 2.4.1. Relative Frequency of Citation (RFC)

RFC determined the agreement among the informants on the use of medicinal plants. Furthermore, this index shows the local importance of each species. The value of RFC for species is based on the citing percentage of informants for that particular plant species [19]. The RFC was calculated using following formula:

(1) $$\begin{array}{c} \text{RFC}=\frac{\text{FC}}{N}\left(0<\text{RFC}<1\right), \end{array}$$

where “FC” is the number of informants mentioned the species while “*N*” indicates the total number of informants participated in the survey.

#### 2.4.2. Use Value (UV)

UV is a good indicator to estimate all the possible uses of a plant species without considering its RFC. It demonstrates the relative importance of plant species known locally, considering the number of uses mentioned by an informant. High values (>1∼) indicate the various use reports for a plant while near zero values highlight the less use reports. However, UV does not distinguish whether a plant is used for single or multiple purposes [20].

This was calculated by following formula:

(2) $$\begin{array}{c} \text{UV}=\sum \frac{Ui}{N}, \end{array}$$

where “*Ui*” is the total number of uses mentioned by each informant for a given species “*i*” and “*N*” is the total number of informants participated in the survey.

#### 2.4.3. Factor Informant Consensus (FIC)

FIC indicates the homogeneity of the information on the use of plants to treat the different types of beauty issues. Higher FIC values (higher when closer to 1) describe the well-defined selection criterion, and/or information is exchanged between informants. If informants do not exchange information about plants' uses, FIC values will be near to zero [21].

FIC was calculated as follows:

(3) $$\begin{array}{c} \text{FIC}=\frac{\left(\text{nur}-\text{nt}\right)}{\left(\text{nur}-1\right)}, \end{array}$$

where “nur” depicts the number of use citations in each category while “nt” indicates the number of used taxa.

#### 2.4.4. Relative Importance (RI)

The following formula was used to calculate the RI:

(4) $$\begin{array}{c} \text{RI}=\text{NUC}+\text{NT,} \end{array}$$

where NUC = number of use categories of a given species/total number of use categories of the most versatile species. NT = types of uses attributed to a given species/total number of types of uses attributed to the most important taxon.

#### 2.4.5. Statistical Analysis

Descriptive statistics were used to present the collected data of the survey. Correlation analysis based on simple linear regression between RFC and UV, RFC and RI, and UV and RI was performed. Statistical significance was set at 5%. The programmes used were Microsoft Excel 2016 and Minitab 17.

## 3. Results and Discussion

### 3.1. General Analysis

#### 3.1.1. Demographic Information of the Informants

A total 120 of informants were interviewed and categorized into different demographic categories as listed in Table 3. There were 43.33% male informants and 56.67% female informants. Most informants were in the 41–50 age group followed by 51–60, 30–40, and 60 < age groups. It was reported as 52.5%, 26.67%, 14.17%, and 6.67%, respectively. On the basis on experience, 37.5% of informants had more than 20 years' experience while 10.00% of informants had less than 5 years' experience. And 30.83% and 21.67% of informants had the experience between 11–20 and 5–10 years, respectively. 50.83% of informants had a bachelor's degree whereas 24.17% had a bachelor's degree with postgraduate education. Furthermore, 25.00% of informants had the inherited knowledge from their families which is known as indigenous knowledge.

#### 3.1.2. Plant Families

A total of 133 different plant species belonging to 64 families were utilized in cosmetic treatments. Of these, 72 plant species are currently used in commercialized herbal cosmetic products in Sri Lanka [22]. The most dominant family was reported as family Fabaceae (21 species, 16%). This was followed by Rutaceae (8 species, 6%); Malvaceae and Zingiberaceae (6 species per each, 5%); Rubiaceae (5 species, 4%); Convolvulaceae, Lamiaceae, and Myrtaceae (4 species per each, 3%); Acanthaceae, Apocynaceae, Arecaceae, Combretaceae, Cucurbitaceae, and Lecythidaceae (3 species per each, 2%); Asteraceae, Celastraceae, Clusiaceae, Oleaceae, Poaceae, Sapindaceae, and Solanaceae (2 species per each, 2%); and one species each for the rest of the families. Table 2 lists the medicinal plants used in cosmetic treatments by traditional and Ayurveda practitioners of Sri Lanka. Moreover, the greatest utilization of medicinal plants in family Fabaceae under different disciplines such as Ayurveda, traditional systems of medicine, and medicinal plant-related industries in Sri Lanka has also been reported by Napagoda et al. [4], Nirmalan [9], Kankanamalage et al. [23], and Dharmadasa et al. [24].

#### 3.1.3. Different Plant Parts Used in Cosmetic Treatments

**Figure 2:**
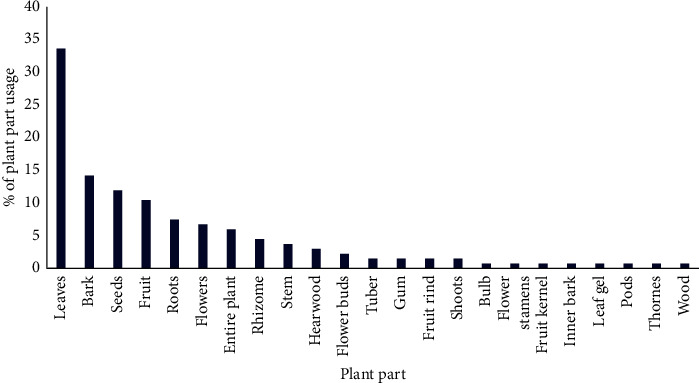
Different parts of the plants used in cosmetic treatments of Sri Lanka.

As shown in Figure 2, a wide range of plant parts were used for cosmetic treatments in Sri Lanka. Identified plant parts were described as leaves, bark, seeds, fruits, roots, flowers, rhizome, stem, heartwood, flower buds, tuber, gum, fruit rind, shoots, bulb, flower stamens, fruit kernel, inner bark, leaf gel, pods, thorns, and wood. The most utilized plant part was stated as leaves (34%). This was followed by bark (14%), seeds (12%), fruit (11%), roots (8%), flowers (7%), rhizome (5%), stem (4%), heartwood (3%), flower buds (2%), tuber, gum, fruit rind, shoots (2% per each), bulb, flower stamens, fruit kernel, inner bark, leaf gel, pods, thorn, and wood (1% per each), respectively. However, usage of entire plant was recorded as 6%. In line with the studies of Napagoda et al. [4], Kumarasinghe [7], Nirmalan [9], Kankanamalage et al. [23], and Dharmadasa et al. [24], leaves were the commonest part of plants used in different treatments of Ayurveda, traditional systems of medicine, and medicinal plant-based industries in Sri Lanka. Moreover, Kumarasinghe, [7] stated that availability of most effective ingredients in leaves results in the highest usage. However, more scientific studies related to this area are required. As other reasons, Dharmadasa et al. [24] highlight availability in large quantities, easy accessibility, and cheaper cost for the abundance usage of leaves. These are further agreement with scientific studies conducted by Miraldi et al. [25], Ghorbani et al. [26] and Mowobi et al. [27] from other countries.

#### 3.1.4. Plants Used in Different Cosmetic Treatments

**Figure 3:**
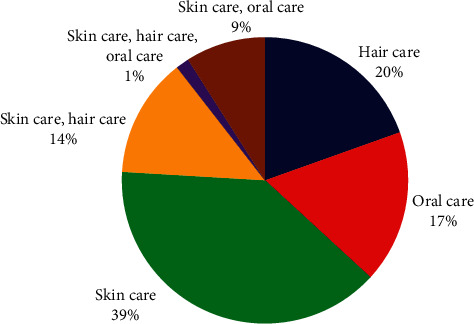
Plant usage in different cosmetic treatments.

Plant species used for cosmetic treatments were classified into three main categories called skin care, hair care, and oral care. Out of 133 plants, the highest numbers of plants were used in skin care treatments. As shown in Figure 3, it was reported as 52 plant species (39%). 26 plant species (20%) and 23 plant species (17%) were used for hair care and oral care treatments, respectively. Moreover, usages of 18 plant species (14%) were reported in both skin and hair care treatments while usages of 12 plant species (9%) were recorded in both skin and oral care treatments. However, the usage of *Myristica fragrans* Houtt. and *Kaempferia galanga* L. was stated in all three cosmetic treatments. Furthermore, research study conducted by Nirmalan [9] on cosmetic perspectives of ethnobotany in Northern part of Sri Lanka confirms the higher usage of local plants for skin care followed by hair care.

According to the market research report on “Organic Personal Care and Cosmetic Products Market by Product Type (Skin Care, Hair Care, Oral Care, and Makeup Cosmetics) and by Distribution Channel (Retail Sale and Online Sale), Global Opportunity Analysis and Industry Forecast, 2015–2022” [28], natural and organic skin care product category is the top billing in the global organic beauty market, and it is expected to emerge as the most attractive segment with 30.9% share by 2024, followed by hair care. Furthermore, research report on “Herbal Beauty Market: Global Industry Analysis and Opportunity Assessment 2015–2025” [29], reports North America and Europe hold the largest market share for herbal sun care and herbal skin care products at present.

#### 3.1.5. Different Mode of Applications in Cosmetic Treatments

**Figure 4:**
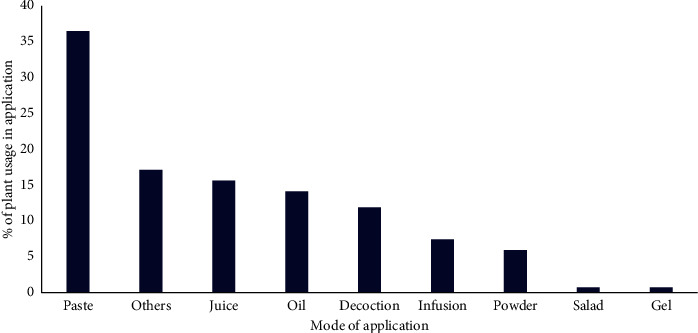
Different modes of applications.

Medicinal plants used in folk herbal remedies were prepared and administered in various forms. Results revealed that there were 08 types of different applications in cosmetic treatments (Figure 4). These include paste, juice, oil, decoction, powder, infusion, gel, and salad. Number of plants used in each application was reported as 49 plant species (37%) as paste, 21 plant species (16%) as juice, 19 plant species (14%) as oil, 16 plant species (12%) as decoction, 8 plant species (6%) as powder, 10 plant species (8%) as infusion, and 01 plant species per each (1%) as gel and salad. However, 10 plant species were reported without specific mode of application while 12 plants were recorded in usage as fragrant agents. Therefore, these 22 plants were included under “others” category. The majority of herbal recipes were prepared using water as the medium. However, honey, coconut oil, and sesame oil were the most commonly used adjuvants in cosmetic treatments. For example, oil prepared by boiling leaves and flowers of *Hibiscus rosa-sinensis* L. with coconut oil or sesame oil is popular application for promoting healthy hair growth, preventing early grey hair, hair fall, and dandruff. Furthermore, *Cinnamomum verum* J.Presl powder mixed with honey is commonly used to treat pimples.

### 3.2. Quantitative Analysis of Data

#### 3.2.1. Relative Frequency of Citation (RFC)

Relative frequency of citation was calculated to identify the most common occurring medicinal plants used for topical cosmetic treatments. Based on RFC values, *Aloe vera* (L.) Burm.f. reported the highest RFC value (0.83) while *Santalum album* L. *Coscinium fenestratum* (Goetgh.) Colebr. and Azadirachta indica A.Juss. showed the second highest RFC value (0.74). In addition, *Kokoona zeylanica*, *Tephrosia purpurea (L*.) Pers., *Theobroma cacao* L., and *Psidium guajava* L. signified 0.73 RFC value. *Terminalia bellirica* (Gaertn.) Roxb., *Elaeocarpus serratus* L., *Cymbopogon citratus* (DC.) Stapf, *Centella asiatica* (L.) Urb, *Terminalia chebula* Retz., *Sesamum indicum* L., *Curcuma longa* L., *Vetiveria zizanioides* (L) Nash, *Myristica fragrans* Houtt., *Mimusops elengi* L., *Citrus limon* (L.) Osbeck, *Eclipta prostrata* (L.)L, *Pterocarpus santalinus* L.f, *Curcuma aromatica* Salisb., *Hemidesmus indicus* (L.) R. Br. ex Schult., *Cucumis sativus* L., *Dillenia retusa* Thunb., *Trigonella foenum-graecum* L., *Cocos nucifera* L., *Acorus calamus* L., *Carica papaya* L., *Asparagus racemosus* Willd., *Indigofera tinctoria* L., and *Cinnamomum verum* J. Presl were other highly cited medicinal plants by traditional practitioners and Ayurveda physicians. The higher RFC values of medicinal plants depict that these plants are well known to the maximum number of informants in terms of cosmetic treatments [19]. However, the least RFC value was reported as 0.01 and it was represented by 24 medicinal plants. Those were *Vernonia anthelmintica* (L.) Willd., *Cuscuta chinensis* Lam., *Costus speciosus* (Koenig) Smith, *Cucumis melo* L., *Diospyros malabarica* (Desr.) Kostel., *Acacia concinna* (Willd.) DC., *Acacia nilotica* (L.) Delile, *Crotalaria verrucosa* L., *Pterocarpus marsupium* Roxb., *Saraca asoca* (Roxb.) Willd., *Curculigo orchioides* Gaertn., *Pogostemon heyneanus* Benth., *Premna obtusifolia* R.Br., *Tectona grandis* L.f., *Barringtonia acutangula* (L.) Gaertn., *Careya arborea* Roxb., *Couroupita guianensis* Aubl., *Abutilon indicum* (L.) Sweet, *Geophila repens* (L.) I.M.Johnst., *Oldenlandia corymbosa* L., *Datura metel* L., *Symplocos cochinchinensis* (Lour.) S. Moore, *Leea indica* (Burm.f.) Merr., and *Alpinia malaccensis* (Burm.f.) Roscoe. Unfortunately, other ethnobotanical studies conducted in Sri Lanka have not focused to this index. Therefore, it was not possible to compare data.

#### 3.2.2. Use Value (UV)

This index highlights the connotation between the medicinal plant species and uses allocated to it [19]. Based on the results of the study, UV was greater than 01 in 19 medicinal plant species while 18 medicinal plants reported the least UV value. The highest UV value was recorded from the *Aloe vera* (L.) Burm.f. It was reported as 3.66. This was followed by *Asparagus racemosus* Willd. (2.20), *Santalum album* L. (1.91), *Myristica fragrans* Houtt. (1.53), *Kokoona zeylanica* Thwaites (1.37), *Indigofera tinctoria* L. (1.30), *Citrus aurantifolia* Panzer. (Christm.) Swingle (1.30), *Centella asiatica* (L.) Urb (1.27), *Coscinium fenestratum* (Goetgh.) Colebr. (1.27), *Azadirachta indica* A.Juss (1.22), *Cymbopogon citratus* (DC.) Stapf (1.13), *Terminalia bellirica* (Gaertn.) Roxb. (1.12), *Sesamum indicum* L. (1.12), *Citrus limon* (L.) Osbeck (1.10), *Eclipta prostrata* (L.) L (1.09), *Theobroma cacao* L. (1.09), *Acorus calamus* L. (1.05), *Terminalia chebula* Retz. (1.03), and *Dillenia retusa* Thunb. (1.00). High UVs indicate the frequent usage of these medicinal plant materials for cosmetic treatments by traditional practitioners and Ayurveda physicians with high use reports. Although the UV index was not calculated, ethnobotanical surveys conducted to find out medicinal plants used in anti-inflammatory remedies [4], skin diseases [7], and snake bites treatments [24] of Sri Lanka revealed about greater usage of *Azadirachta indica* A.Juss., in skin diseases treatments, *Citrus aurantifolia* (Christm.) Swingle in snake bites treatments, and *Coscinium fenestratum* (Goetgh.) Colebr. in anti-inflammatory remedies, respectively. According to the survey on medicinal materials used in traditional systems of medicine in Sri Lanka, *Aloe vera* (L.) Burm.f., *Santalum album* L., *Coscinium fenestratum* (Goetgh.) Colebr., and *Acorus calamus* L. have been listed as heavily used medicinal plants in Sri Lanka. Moreover, *Sesamum indicum* L., *Azadirachta indica* A.Juss, *Indigofera tinctoria* L., *Terminalia bellirica* (Gaertn.) Roxb., *Terminalia chebula* Retz., *Asparagus racemosus* Willd., and *Centella asiatica* (L.) Urb have been listed as the most demanded plant materials which show heavy usage in the Sri Lankan traditional systems of medicine [23].

#### 3.2.3. Relative Importance (RI)

There were 12 medicinal plants which recorded the RI value greater than 1. The highest RI value was reported from *Asparagus racemosus* Willd. and *Hibiscus rosa-sinensis* L. It was 1.67. This was followed by *Kaempferia galanga* L. (1.57), *Aloe vera* (L.) Burm.f. (1.52), *Myristica fragrans* Houtt. (1.43), *Citrus aurantifolia* Panzer. (Christm.) Swingle (1.24), *Acorus calamus* L. (1.10), *Eclipta prostrata* (L.) L (1.10), *Indigofera tinctoria* L. (1.10), *Jasminum grandiflorum* L. (1.10), *Phyllanthus emblica* L. (1.10), and *Morinda citrifolia* L. (1.10). The lowest RI value was 0.48, and it was recorded from 73 medicinal plants. This may possibly be due to the less used categories and a few used attributes of these plants.

#### 3.2.4. Factor Informant Consensus (FIC)

The results of the FIC (Table 4) showed that treatments for wrinkles and foot health improvements (1.00) under the skin care category, treatments for damaged hair and scalp cooling (1.00) under the hair care category, and treatments for malodour of the mouth (0.96) under oral care category had the higher degree of consensus. All these values were comparatively high, thus indicating good agreement among traditional practitioners and Ayurveda physicians on the knowledge and consequent use of particular plants or plant materials to treat different beauty issues.

Medicinal plants are used in cosmetic treatments for various reasons. The close analysis of the survey revealed the medicinal plant usage in 16 skin-related issues, 9 hair-related issues, and 2 major oral-related issues. The majority of medicinal plants used in skin care treatments were used to improve skin complexion. It was reported as 25 medicinal plants. Treatment for pimples was the second largest treatment segment under the skin care which reported 23 medicinal plants in usage. Other than these main two treatments, herbal preparations were used to treat for freckles (13 plants), skin discoloration (9 plants), healing (8 plants), aging (7 plants) and acne, skin dryness, exfoliating and soften the skin (4 plants per each), malodour of the body, cleansing, skin health, and whitening (2 plants per each). *Camellia sinensis* (L.) Kuntze is used to treat for skin wrinkles. Furthermore, *Ipomoea pes-caprae* (L.) R. Br. is utilized to improve foot health. The most of hair care treatments were done for improving the healthy hair followed by dandruff treatments. It was reported as usage of 20 and 11 medicinal plants, respectively. Moreover, treatments were recorded for pediculosis (9 plants), cleansing and hair fallen (8 plants per each), grey hair (6 plants), and improving hair color (4 plants). Moreover, *Centella asiatica* (L.) Urb is used to treat damaged hair whilst *Jasminum grandiflorum* L. plant extracted oil is used for scalp cooling. Majority of treatments under oral care were reported for improving oral hygiene. Most of treatments of oral care were prepared as mouth wash. 34 medicinal plants used to improve oral hygiene were reported. Furthermore, *Cryptolepis dubia* (Burm.f.) M.R. Almeida, *Saraca asoca* (Roxb.) Willd., and *Hibiscus abelmoschus* L. are used to treat particularly for malodour of the mouth. In addition, 12 medicinal plant species which used as fragrant agent were also recorded.

#### 3.2.5. Correlation Analysis

**Figure 5:**
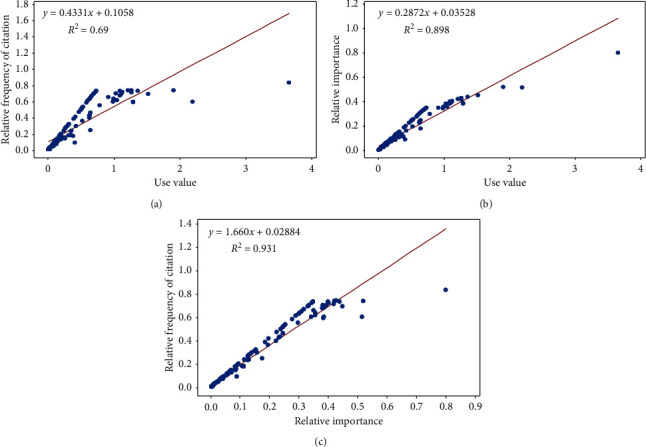
Correlation among different quantitative measures. (a) Correlation between RFC and UV. (b) Correlation between RI and UV. (c) Correlation between RFC and RI.

Correlations among different quantitative measures are indicated in Figure 5. RFC was moderately correlated with UV (Figure 5(a), *R*^2^ = 0.69, *P*=0.0001) while high correlation was reported between RI and UV (Figure 5(b), *R*^2^ = 0.898, *P*=0.0001) and RFC and RI (Figure 5(c), *R*^2^ = 0.931, *P*=0.0001), giving good linearity between variables. Therefore, this is proven that identified plant species significantly have the same importance irrespective to the methodology employed.

## 4. Conclusion

To our knowledge, this was the first survey carried out to identify cosmetic potential medicinal plants throughout the island, Sri Lanka. High RFC, UV, and FIC values of medicinal plants from the current study indicate the better consensus among traditional practitioners and Ayurveda physicians about the usage of medicinal plants or plant materials for cosmetic treatments. Neither toxicity nor toxicity removal methods of the identified plants or plant materials were mentioned during the discussions with Ayurveda physicians and traditional practitioners. Therefore, further studies on toxicity and safety of identified plants or plant materials are paramount for herbal cosmetic product development in the future. Assembling of multidisciplinary cooperation of botanists, chemists, toxicologists, researchers, and biologists is suggested to analyze the interesting functional properties, safety, efficacy, and effectiveness of documented cosmetic potential medicinal plants. The present ethnopharmacological study on medicinal plants used for topical cosmetic treatments by traditional practitioners and Ayurveda physicians of Sri Lanka will contribute to preserve the traditional knowledge of medicinal plants. Also, this information could ultimately be utilized for the development of the herbal cosmetic industry in Sri Lanka.

## Figures and Tables

**Table 1:** Distribution of respondents in 9 provinces of Sri Lanka.

Province	Respondents	
	Ayurveda Physicians	Traditional Practitioners
Northern	9	2
North Western	11	5
Western	16	3
Central	9	3
Uva	8	1
Sabaragamuwa	9	4
Eastern	7	1
Southern	11	4
North Central	10	7
Total	**90**	**30**
	**120**	

**Table 2:** Medicinal plants used in topical cosmetic treatments of Sri Lanka.

No.	Family	Scientific name	English name	Vernacular name	Plant part	Potential usage	Treatment/s for	Mode of application	RFC	UV	RI
1	Acanthaceae	*Barleria lupulina* Lindl.	Hophead Philippine violet	Ranwan katu karandu	Leaves	S	PI	JU	0.09	0.09	0.04
2		*Barleria prionitis* L.	Crossandra	Katu karandu	Leaves	S, H	SH, GH	JU	0.11	0.16	0.06
3		*Justicia adhatoda* L.^*∗*^	Malabar nut	Adhatoda	Leaves	O	OH	OT	0.42	0.42	0.20
4	Acoraceae	*Acorus calamus* L.	Sweet flag	Wada-kaha	Rhizome	S, H	PE, SC, FA	PA	0.62	1.05	0.36
5	Amaranthaceae	*Alternanthera sessilis* (L.) R.Br. ex DC^*∗*^	Sessile joyweed	Mukunuwenna	Leaves, stem	H	HH	JU	0.30	0.30	0.14
6	Amaryllidaceae	*Allium sativum* L.	Garlic	Sudulunu	Bulb	S	PI	PA	0.31	0.32	0.15
7	Annonaceae	*Annona muricata* L.	Prickly custard apple	Katu-anoda	Tender leaves	H	PE	JU	0.28	0.28	0.13
8	Apiaceae	*Centella asiatica* (L.) Urb^*∗*^	Indian pennywort	Gotukola	Entire plant	S, H	DH, HE	PA	0.71	1.27	0.42
9	Apocynaceae	*Cryptolepis dubia* (Burm.f.) M.R.Almeida		Wel rukkattana	Leaves, bark, flowers	S, O	MM, MB	DE	0.08	0.12	0.04
10		*Hemidesmus indicus* (L.) R. Br. ex Schult.^*∗*^	Indian sarsaparilla	Iramusu	Leaves	S, H	HF, SC	JU	0.66	0.92	0.35
11		*Holarrhena antidysenterica* (Roxb.) Wall.	Kurchi	Kelinda	Bark	O	OH	DE	0.20	0.20	0.10
12	Arecaceae	*Areca catechu* L.	Areca nut	Puwak	Flowers	O	OH	DE	0.08	0.08	0.04
13		*Caryota urens* L.	Wine palm	Kithul	Bark, tender flowers	H	HH	OT	0.06	0.07	0.03
14		*Cocos nucifera* L.^*∗*^	King coconut/Pol	Thambili/Coconut	Fruit kernel	H	HH	OI	0.63	0.63	0.30
15	Asparagaceae	*Asparagus racemosus* Willd.^*∗*^	Wild asparagus	Hathawariya	Entire plant	S, H	HC, HH. HF, GH, AG, SC, FR	JU	0.60	2.20	0.51
16	Asphodelaceae	*Aloe vera* (L.) Burm.f.^*∗*^	Aloe plant	Komarika	Leaf gel	S, H	FR, PI, HF, DN, AG, EX	GE	0.83	3.66	0.80
17	Asteraceae	*Eclipta prostrata* (L.) L^*∗*^	False daisy	Keekirindiya	Leaves	S, H	HH, SD, AG	OI, JU	0.68	1.09	0.38
18		*Vernonia anthelmintica* (L.) Willd.	Purple fleebane	Sanninayam	Seeds	S, H	FR, PE	PA	0.01	0.02	0.01
19	Berberidaceae	*Berberis aristata* DC.	Indian barberry	Daruharidra	Bark	O	OH	DE	0.02	0.02	0.01
20	Bombacaceae	*Bombax ceiba* L.	Red silk cotton tree	Imbul	Thrones, seeds	S	SC, FR, PI	PA, OI	0.05	0.08	0.03
21	Boraginaceae	*Heliotropium indicum* L.	Indian heliotrope	Ethhoda	Leaves	S	PI	JU	0.03	0.03	0.01
22	Brassicaceae	*Brassica juncea* (L.) Czern	Indian mustard	Aba	Seeds	S	SS, SC	OI	0.36	0.53	0.20
23	Calophyllaceae	*Mesua ferrea* L.^*∗*^	Iron wood	Na	Flower stamens	S	MB, FR, SC, FA	PA	0.24	0.36	0.13
24	Cannabaceae	*Celtis timorensis* Span.	Stinkwood	Gurenda/Burenda	Wood	S	DR, SC	PA	0.02	0.03	0.01
25	Caricaceae	*Carica papaya* L.^*∗*^	Papaya	Gaslabu	Ripen fruit	S	FR	PA	0.61	0.61	0.29
											
26	Celastraceae	*Celastrus paniculatus* Willd.	Black oil plant	Duhudu	Bark	H	PE	PA	0.03	0.03	0.01
27		*Kokoona zeylanica* Thwaites^*∗*^		Kokun	Bark	S	SC, PI	PO	0.73	1.37	0.44
28	Clusiaceae	*Garcinia mangostana* L.	Mangosteen	Mangus	Bark	O	OH	DE	0.09	0.09	0.04
29		*Garcinia quaesita* Pierre.^*∗*^	Red mango	Goraka	Fruit rind, bark	O	OH	OT	0.29	0.28	0.14
30	Combretaceae	*Terminalia arjuna* (Roxb.) Wight & Arn.	Arjuna myrobalan	Kumbuk	Bark	S, O	PI, OH	PA	0.18	0.19	0.09
31		*Terminalia bellirica* (Gaertn.) Roxb.^*∗*^	Beleric myrobalan	Bulu	Seeds	H	HC, HF	OI	0.72	1.12	0.40
32		*Terminalia chebula* Retz.^*∗*^	Myrabalans	Aralu	Fruit	S, O	OH, AG	IN	0.70	1.03	0.38
33	Convolvulaceae	*Argyreia populifolia* Choisy	Sri Lankan elephant creeper	Giritilla	Shoots	O	OH	JU	0.05	0.05	0.02
34		*Cuscuta chinensis* Lam.	Dodder	Agamula nathi wal	Entire plant	H	HC, HF, DN	IN	0.01	0.03	0.01
35		*Evolvulus alsinoides* (L.) L.	Slender dwarf morning- glory	Vishnukranthi	Entire plant	H	HH	OI	0.07	0.08	0.04
36		*Ipomoea pes-caprae* (L.) R. Br.	Goats foot creeper	Binthamburu	Leaves	S	FH	DE	0.02	0.02	0.01
37	Costaceae	*Costus speciosus* (Koenig) Smith	Crape ginger	Thebu	Bark	S	FR	OT	0.01	0.01	0.00
38	Cucurbitaceae	*Cucumis melo L*.	Bitter cucumber	Gon kakiri	Fruit	S	PI	PA	0.01	0.01	0.00
39		*Cucumis sativus* L.^*∗*^	Cucumber	Pipingna	Fruit	S	HE	SA	0.65	0.65	0.31
40		*Trichosanthes cucumerina* L.	Wild sankegourd	Dummalla	Leaves	H	GH	DE	0.03	0.03	0.01
41	Cyperaceae	*Cyperus rotundus* L.^*∗*^	Nutgrass	Kalanduru	Tuber	S	AC	PA	0.54	0.53	0.26
42	Dilleniaceae	*Dillenia retusa* Thunb.^*∗*^		Godapara	Fruit	H	HH, HCL	PA	0.64	1.00	0.36
43	Ebenaceae	*Diospyros malabarica* (Desr.) Kostel.	Riber ebony	Timbiri	Fruit	O	OH	DE	0.01	0.01	0.00
44	Elaeocarpaceae	*Elaeocarpus serratus* L.^*∗*^	Wild olive	Weralu	Leaves	H	HCL	IN	0.72	0.73	0.34
45	Euphorbiaceae	*Jatropha curcas* L	Purging nut	Rata endaru	Bark	O	OH	DE	0.08	0.08	0.04
46	Fabaceae	*Abrus precatorius* L.	Wild liquorice	Olinda	Seeds	S	AC	PA	0.27	0.27	0.13
47		*Acacia concinna* (Willd.) DC.		Seenidda	Pods	H	HH, DN, PE, HCL	PO	0.01	0.03	0.01
48		*Acacia nilotica (*L.) Delile	Gum Arabic tree	Babbula	Leaves, bark, stem	S, O	DR, OH	PA	0.01	0.02	0.01
49		*Adenanthera pavonina* L.^*∗*^	Red lucky seed	Madatiya	Leaves	S	PI, SC	JU	0.30	0.43	0.16
50		*Caesalpinia bonduc* (L.) Roxb.	Molucca bean	Kumburu	Seeds	S, O	PI, OH	OI, PO	0.02	0.03	0.01
51		*Caesalpinia sappan *L.	Sappanwood	Patagi	Heartwood	S	WH, AC	PO	0.05	0.08	0.03
											
52		*Crotalaria verrucosa* L.	Blue rattle weed	Nil-adanahiriya	Leaves, shoots	O	OH	IN	0.01	0.01	0.00
53		*Entada rheedii* Spreng.	Elephant creepes mackay	Pus-wel	Seeds, bark, stem	S, H	PI, HCL	PA	0.04	0.03	0.02
54		*Glycyrrhiza glabra *L.^*∗*^	Liqourice	Walmee	Roots	S	SC	OT	0.52	0.52	0.25
55		*Indigofera tinctoria* L.^*∗*^	Indigo	Nil-awari	Leaves	S, H	SC, HF, HH	OI, PA	0.60	1.30	0.39
56		*Mimosa pudica* L.	Sensitive plant	Nidi-kumba	Entire plant	S, O	HE, OH	PA, DE	0.10	0.14	0.05
57		*Pongamia pinnata* (L.) Pierre^*∗*^	Indian beech	Karanda	Seeds	S	SD	OI	0.12	0.12	0.06
58		*Pterocarpus marsupium* Roxb.	Indian kino tree	Gammalu	Gum	O	OH	OT	0.01	0.01	0.00
59		*Pterocarpus santalinus* L.f.^*∗*^	Red sandalwood	Rath-handun	Heartwood	S	PI	PA	0.67	0.67	0.32
60		*Saraca asoca *(Roxb.) Willd.	Ashoka tree	Asoka	Bark	O	MM, OH	PO	0.01	0.02	0.01
61		*Senna alata* (L.) Roxb.^*∗*^	Candle bush	Eththora	Leaves	S	SD	PA	0.54	0.53	0.26
62		*Senna alexandrina* Mill.	Indian senna	Senehekola	Leaves	H	DN, HH	DE	0.02	0.03	0.01
63		*Senna auriculata* (L.) Roxb.^*∗*^	Tanner's cassia	Ranawara	Flowers	S	SC	PA	0.15	0.15	0.07
64		*Sesbania grandiflora* (L.) Pers.^*∗*^	Vegetable hummingbird	Kathurumurunga	Leaves	H	HH	OI	0.47	0.48	0.23
65		*Tephrosia purpurea* (L.) Pers.^*∗*^	Purple tephrosia	Kathurupila	Roots	O	OH	OT	0.73	0.73	0.35
66		*Trigonella foenum-graecum* L.^*∗*^	Fenugreek	Uluhal	Seeds	H	DN	IN	0.64	0.64	0.30
67	Hypoxidaceae	*Curculigo orchioides* Gaertn.	Black musale	Binthal	Tuber	S	SC	PA	0.01	0.01	0.00
68	Lamiaceae	*Ocimum tenuiflorum* L.^*∗*^	Holy basil	Heen maduruthala	Leaves	S	FA, PI, FR	OI, JU	0.19	0.32	0.11
69		*Pogostemon heyneanus* Benth.	Patchouli	Gas-kollankola	Leaves	O	OH	IN	0.01	0.01	0.00
70		*Premna obtusifolia* R.Br.		Heen midi	Leaves	H	HCL	JU	0.01	0.01	0.00
71		*Tectona grandis* L.f.	Teak	Thekka	Fruit	H	HH	PO	0.01	0.01	0.00
72	Lauraceae	*Cinnamomum verum* J.Presl^*∗*^	Ceylon cinnamom	Kurundu	Inner bark	S, O	PI, OH	PA	0.60	0.99	0.34
73	Lecythidaceae	*Barringtonia acutangula* (L.) Gaertn.	Indian oak	Midella	Leaves, bark, flower	O	OH	DE	0.01	0.01	0.00
74		*Careya arborea* Roxb.	Patana oak	Kahata	Bark gum	S	SS	OT	0.01	0.01	0.00
75		*Couroupita guianensis* Aubl.	Cannon ball tree	Sal	Leaves	S	HE	OT	0.01	0.01	0.00
76	Lythraceae	*Lawsonia inermis* L.^*∗*^	Henna	Marathondi	Leaves	H	GH, HH	IN	0.44	0.64	0.24
77		*Punica granatum* L.^*∗*^	Pomegranate	Delum	Leaves, fruit rind, roots	S, O	OH	DE	0.07	0.08	0.04
78	Magnoliaceae	*Michelia champaca* var. blumei Moritzi	Champak	Gini sapu	Flowers	S	FA, HE	OI	0.05	0.08	0.03
79	Malvaceae	*Abutilon indicum* (L.) Sweet	Country mallow	Behethanoda	Leaves	O	OH	PA	0.01	0.01	0.00
80		*Gossypium arboreum* L.	Cotton	Kapu	Leaves	S	HE	OT	0.06	0.07	0.03
81		*Hibiscus abelmoschus* L.^*∗*^	Musk mallow	Kapukinissa	Seeds	S, O	MM, FA	PA	0.40	0.63	0.22
82		*Hibiscus rosa-sinensis* L.^*∗*^	Shoe flower	Pokuru wada	Leaves, flowers	S, H	HH, GH, HF, DN, SD, PI, FR	OI, PA	0.09	0.41	0.09
83		*Sida cordata (Burm*.*f*.) Borss. Waalk^*∗*^	Heart leaf sida	Wel bebila	Leaves, stem	S, H	HH, PI	PA	0.16	0.23	0.08
84		*Theobroma cacao* L.^*∗*^	Coco	Kokova	Seeds	S	DR, SS	OT	0.73	1.09	0.40
85	Meliaceae	*Azadirachta indica* A.Juss^*∗*^	Neem	Kohomba	Leaves, roots	S, O	PI, OH	PA, DE	0.74	1.22	0.42
86	Menispermaceae	*Coscinium fenestratum* (Goetgh.) Colebr.^*∗*^	Calumba wood	Weniwel	Stem	S	EX, AC	PA	0.74	1.27	0.43
87	Moraceae	*Fiscus racemosa* L.^*∗*^	Country fig	Attikka	Leaves	S	FR, SD	PA	0.46	0.65	0.25
88	Moringaceae	*Moringa oleifera* Lam.^*∗*^	Drumstick tree	Murunga	Leaves	S	PI, FR, SS, SH	JU, PA	0.07	0.13	0.04
89	Myristicaceae	*Myristica fragrans* Houtt.^*∗*^	Nutmeg	Sadhikka	Seeds	S, H, O	PE, HCL, OH	OI	0.69	1.53	0.45
90	Myrtaceae	*Melaleuca leucadendra* (L.) L.^*∗*^	Cajuput tree	Lothsumbulu	Bark	S	SC	OT	0.52	0.52	0.25
91		*Psidium guajava* L.^*∗*^	Guava	Pera	Leaves	H	HH	JU	0.73	0.73	0.35
92		*Syzygium aomaricum* (L.) Merr. & L.M.Perry^*∗*^	Clove	Karabu	Flower buds	O	OH	OT	0.09	0.09	0.04
93		*Syzygium cumini* (L.) Skeels	Indian black berry	Madan	Bark	O	OH	OT	0.14	0.14	0.07
94	Nymphaeaceae	*Nymphaea nouchali Burm*.*f*^*∗*^	Blue water lily	Nil manel	Flowers	H	HC	PA	0.32	0.33	0.15
95	Oleaceae	*Jasminum grandiflorum* L.^*∗*^	Jasmine	Samanpichcha	Flowers, leaves, roots	S, H	SD, FA, SCO	PA, OI	0.19	0.36	0.11
96		*Jasminum multiflorum* (Burm.f.) Andrews	Sambac Jasmine	Geta pichcha	Flower buds	S	SC	PA	0.06	0.07	0.03
97	Oxalidaceae	*Averrhoa bilimbi L*.	Bilimbi	Bilin	Leaves	S, H	PI, PE	JU	0.56	0.79	0.30
98	Pedaliaceae	*Sesamum indicum L*.^*∗*^	Gingelly	Thel-thala	Seeds	S, H	HH, HE	OT	0.70	1.12	0.39
99	Phyllanthaceae	*Phyllanthus emblica L*.^*∗*^	Emblic myrobalan	Nelli	Leaves, fruit	S, H	SC, HF, GH,	OT	0.18	0.38	0.11
100	Pinaceae	*Cedrus deodara* (Roxb. ex D.Don) G.Don^*∗*^	Himalayan cedar	Dewadara	Heartwood	S	FA	OT	0.24	0.24	0.11
101	Piperaceae	*Piper nigrum* L.^*∗*^	Pepper	Gammiris	Seeds	S, O	FA	OT	0.58	0.58	0.28
102	Plantaginaceae	*Bacopa monnieri* (L.) Wettst.^*∗*^	Thyme leaved gratiola	Lunuwila	Entire plant	S, H	AG, DN	OT	0.16	0.25	0.09
103	Poaceae	*Cymbopogon citratus (DC*.) Stapf^*∗*^	Lemon grass	Sera	Entire plant	H	PE, DN, FA	OI	0.72	1.13	0.40
104		*Vetiveria zizanioides* (L) Nash^*∗*^	Khas-khas	Sawandara	Roots	S	FA	OI, PA	0.70	0.70	0.33
105	Ponterderiaceae	*Monochoria vaginalis* (Burm.f.) C. Presl^*∗*^	Oval leafed pondweed	Diyahabarala	Roots	O	OH	IN	0.18	0.18	0.08
106	Rubiaceae	*Coffea arabica *L.^*∗*^	Coffee	Kopi	Seeds	S	EX	PO	0.12	0.12	0.06
107		*Geophila repens *(L.) I.M.Johnst.		Kothurubedda	Leaves	S	SD	PA	0.01	0.01	0.00
108		*Morinda citrifolia* L.	Indian mulberry	Ahu	Leaves, fruit	S, O	OH, EX, HE	JU, PA	0.15	0.26	0.09
109		*Oldenlandia corymbosa *L.	Diamond flower	Pathpdagam	Entire plant	S	PI	OT	0.01	0.01	0.00
110		*Rubia cordifolia* L.^*∗*^	Heart leaved madder	Velmadata	Roots	S	FR, SD	PA	0.43	0.63	0.23
111	Rutaceae	*Acronychia pedunculata* (L.) Miq.^*∗*^	Claw flowered laurel	Ankenda	Leaves	S	PI	PA	0.39	0.39	0.19
112		*Aegle marmelos* (L.) Correa^*∗*^	Bael fruit tree	Beli	Ripen fruit	S	SC	PA	0.53	0.53	0.25
113		*Citrus aurantifolia* Panzer. (Christm.) Swingle^*∗*^	True lime	Dehi	Fruit	S, H	HCL, SCL, DN, PE	In, PA	0.59	1.30	0.38
114		*Citrus hystrix* DC.	Kaffir lime	Gada dehi	Fruit	H	DN, FA	JU	0.13	0.20	0.07
115		*Citrus limon (*L.) Osbeck^*∗*^	Lemon	Lemon	Fruit	S	SCL, DR	JU	0.69	1.10	0.39
116		*Melicope lunu-ankenda* (Gaertn.) T.G. Hartley		Lunu-ankenda	Leaves	S	SC	PA	0.02	0.02	0.01
117		*Murraya koenigii (*L.) Spreng.	Curry leaf	Karapincha	Leaves	H	HH	JU	0.50	0.50	0.24
118		*Ruta graveolens* L.^*∗*^	Garden rue	Aruda	Leaves	H	HH	OI	0.52	0.52	0.25
119	Santalaceae	*Santalum album* L.^*∗*^	Sandalwood	Sudu handun	Heartwood	S	PI, FR, SC, AG	PA	0.74	1.91	0.52
120	Sapindaceae	*Sapindus mukorossi* Gaertn.^*∗*^	Soap nut	Gas penela	Fruit	H	HCL	PA	0.07	0.08	0.04
121		*Schleichera oleosa* (Lour.) Merr.^*∗*^	Ceylon oak	Kon	Seeds	H	HH	OI	0.19	0.19	0.09
122	Sapotaceae	*Mimusops elengi* L.^*∗*^	Bullet wood tree	Munamal	Bark	O	OH	DE	0.69	0.69	0.33
123	Solanaceae	*Datura metel* L.	Datura	Attana	Roots	O	OH	PO	0.01	0.01	0.00
124		*Withania somnifera *(L.) Dunal^*∗*^	Indian ginseng	Amukkra	Roots	S	AG	OT	0.04	0.03	0.02
125	Symplocaceae	*Symplocos cochinchinensis* (Lour.) S. Moore		Bombu	Bark	O	OH	DE	0.01	0.01	0.00
126	Theaceae	*Camellia sinensis* (L.) Kuntze^*∗*^	Tea	Thae	Tender leaves	S	WR	OT	0.13	0.13	0.06
127	Vitaceae	*Leea indica (Burm*.*f*.) Merr.	Bandicoot berry	Gurulla	Leaves	S	SD, SC	PA	0.01	0.02	0.01
128	Zingiberacea	*Alpinia malaccensis (Burm*.*f*.) Roscoe^*∗*^		Rankihiriya	Flower buds	O	OH	JU	0.01	0.01	0.00
129		*Curcuma aromatic* Salisb.^*∗*^	Wild turmeric	Kasthuri kaha	Rhizome	S	SC	PA	0.67	0.67	0.32
130		*Curcuma longa* L.^*∗*^	Turmeric	Ath kaha	Rhizome	S	SC	PA	0.70	0.70	0.33
131		*Curcuma zedoaria (christm*.) Roscoe^*∗*^	Zedoary	Haran kaha	Rhizome	S	SC, FA	PA	0.05	0.08	0.03
132		*Kaempferia galanga* L.	Java galanga	Ingurupiyali	Rhizome	S, H, O	OH, SC, DN, WH	In, PA	0.25	0.65	0.18
133		*Zingiber officinale* Roscoe^*∗*^	Ginger	Inguru	Rhizome	S	PI, SC	JU	0.23	0.35	0.13

*Note*. RFC: relative frequency of citation; UV: use value; RI: relative importance; S: skin care; H: hair care; O: oral care; OH: oral hygiene; MM: malodour of the mouth; HH: healthy hair; DN: dandruff; PE: pediculosis; HCL: scalp and hair cleansing; DH: damaged hair; HF: hair fallen; GH: grey hair; HC: hair color; SCO: scalp cooling; SC: skin complexion; PI: pimples; FR: freckles; HE: healing; AG: aging; SD: skin discoloration; AC: acne; DR: skin dryness; EX: exfoliate; SS: soften the skin; INF: infections; MB: malodour of the body; SCL: skin cleansing; SH: skin health; WH: whitening; WR: wrinkles; FA: fragrant agent; FH: foot health; PA: paste; JU: juice; OI: oil; DE: decoction; PO: powder; IN: infusion; GE: gel; SA: salad; OT: others. ^*∗*^ Plants used in commercial herbal cosmetic products in Sri Lanka.

**Table 3:** Demographic information about informants.

Parameter	Percentage (%)
*Gender*	
Male	43.33
Female	56.67

*Age group (years)*	
30–40	14.17
41–50	52.5
51–60	26.67
60<	6.67

*Experience (years)*	
<5	10.00
5–10	21.67
11–20	30.83
20<	37.5

*Education*	
Inherited from family (indigenous)	25.00
Bachelor	50.83
Bachelor + postgraduate	24.17

**Table 4:** Factor informant consensus (FIC) for different beauty issues.

Category	Treatments for	Number of plants	Citations	FIC
Skin care	Improving skin complexion	25	63	0.61
	Pimples	23	80	0.72
	Freckles	13	73	0.83
	Skin discoloration	9	64	0.87
	Healing	8	78	0.91
	Aging	7	72	0.92
	Acne	4	64	0.95
	Skin dryness	4	82	0.96
	Exfoliating	4	18	0.82
	Soften the skin	4	43	0.93
	Malodour of the body	2	29	0.96
	Cleansing	2	82	0.99
	Improving skin health	2	9	0.88
	Whitening	2	30	0.97
	Wrinkles	1	16	1.00
	Improving foot health	1	2	1.00

Hair care	Healthy hair	20	88	0.78
	Dandruff	11	77	0.87
	Pediculosis	9	83	0.90
	Scalp and hair cleansing	8	87	0.92
	Hair fallen	8	72	0.90
	Grey hair	6	52	0.90
	Improving hair color	4	87	0.97
	Damaged hair	1	86	1.00
	Scalp cooling	1	22	1.00

Oral care	Improving oral hygiene	34	88	0.62
	Malodour of the mouth	3	48	0.96

## Data Availability

The data used to support the findings of this study are included within the article.

## Supplementary Materials

Supplementary Materials Supplementary Material 1: the questionnaire which was used to collect the information on plants/plant materials for topical cosmetic treatments by traditional practitioners and Ayurveda physicians.
