# Supplementary material for: Ethnopharmacological Survey on Medicinal Plants Used for Cosmetic Treatments in Traditional and Ayurveda Systems of Medicine in Sri Lanka
Source: Evid Based Complement Alternat Med. 2021 Jun 26;2021:5599654. doi: 10.1155/2021/5599654 (PMC8257331; doi:10.1155/2021/5599654)
Supplement: Supplementary Materials — Supplementary Material 1: the questionnaire which was used to collect the information on plants/plant materials for topical cosmetic treatments by traditional practitioners and Ayurveda physicians. [file 5599654.f1.doc]

**Supplementary Material 1**

**Questionnaire form for ethnobotanical Survey for Identification of Cosmetic Potential Medicinal Plants in Sri Lanka**

**SECTION I**

1. Name: ………………………………………………………………………………….
2. Gender: Male Female

1. Age (years)

30 – 40 41 – 50 51 – 60 60<

1. Experience of the profession (years):

<5 5 -10 11- 20 20 <

1. Education:

Inherited from family (Traditional) Bachelor

Bachelor+ Postgraduate

1. Address: ……………………………………………………………………………….
2. Do you possess a valid registration as a practitioner: ………………………………...
3. If yes, registered no: …………………………………………………………………..
4. Province/ District:

North CentralAnuradhapuraPolonnaruwa

UvaBadullaMonaragala

SabaragamuwaKegalleRatnapura

EasternTrincomaleeBatticaloaAmpara

SouthernHambantotaMataraGalle

NorthernJaffnaKilinochchiMannarMullaitivuVavuniya

North WesternPuttalamKurunegala

WesternGampahaColomboKalutara

CentralMataleKandyNuwaraeliya

**SECTION II**

10) List of medicinal plants and plant part/s used for cosmetics treatments. Please comment about the expected function/s, remedy/s, source and availability of raw materials for cosmetics treatments.

| **Medicinal Plant** | **Plant part** | **Potential usage (Skin care, Hair care, Oral care)** | **Treatments for** | **Mode of application** |
| --- | --- | --- | --- | --- |
|  |  |  |  |  |
|  |  |  |  |  |
|  |  |  |  |  |
|  |  |  |  |  |
|  |  |  |  |  |
|  |  |  |  |  |
|  |  |  |  |  |
|  |  |  |  |  |
|  |  |  |  |  |
|  |  |  |  |  |
|  |  |  |  |  |
|  |  |  |  |  |
|  |  |  |  |  |
|  |  |  |  |  |

11) Add your suggestions for improving the research based on utilization of medicinal plants in cosmetics (future potential research areas).

…………………………………………………………………………………………………………………………………………………………………………………………………………………………………………………………………………………………………………………………………………………………………………………………………………………………………………………………………………………………………………………………………………………………………………………………………………………………………………………………………………………………………………………………………………………………………………………………………………………………………………………………………………………………………………………………………………
